# Evidence-Based Guideline on Laparoscopy in Pregnancy: Commissioned by the British Society for Gynaecological Endoscopy (BSGE) Endorsed by the Royal College of Obstetricians & Gynaecologists (RCOG)

**Published:** 2019-03

**Authors:** E Ball, N Waters, N Cooper, C Talati, R Mallick, S Rabas, A Mukherjee, Y Sri Ranjan, M Thaha, R Doodia, R Keedwell, M Madhra, N Kuruba, R Malhas, E Gaughan, K Tompsett, H Gibson, H Wright, C Gnanachandran, T Hookaway, C Baker, K Murali, D Jurkovic, N Amso, J Clark, S Thangaratinam, T Chalhoub, P Kaloo, E Saridogan

**Affiliations:** Royal London Hospital;; Royal Surrey County Hospital NHS Trust;; Homerton Hospital;; Brighton and Sussex University Hospitals NHS Trust;; Queen’s Hospital London and King George Hospital;; Northwick Park Hospital;; Chelsea and Westminster Hospital;; John Radcliffe Hospital;; Royal Cornwall Hospitals NHS Trust;; Royal Infirmary of Edinburgh;; Norfolk and Norwich University Hospital;; Walsall Manor Hospital;; Rotunda Hospital, Dublin;; Barking, Havering and Redbridge University Hospitals NHS Trust;; North Manchester General Hospital;; Northampton General Hospital;; Royal Gwent Hospital Aneurin Bevan Health Board;; Plymouth Hospitals NHS Trust;; Poole Hospital;; Salisbury District and General Hospital;; University College Hospital;; Cardiff University School of Medicine;; Birmingham Women’s Hospital;; Royal Victoria Infirmary;; Gloucestershire Hospitals NHS Foundation Trust.

**Keywords:** Laparoscopy in pregnancy, Appendicitis in pregnancy, Gallbladder disease / Cholecystitis in pregnancy, Benign adnexal tumours / Ovarian cysts in pregnancy

## Abstract

Laparoscopy is widely utilised to diagnose and treat acute and chronic, gynaecological and general surgical conditions. It has only been in recent years that laparoscopy has become an acceptable surgical alternative to open surgery in pregnancy. To date there is little clinical guidance pertaining to laparoscopic surgery in pregnancy. This is why the BSGE commissioned this guideline. MEDLINE, EMBASE, CINAHL and the Cochrane library were searched up to February 2017 and evidence was collated and graded following the NICE-approved process. The conditions included in this guideline are laparoscopic management of acute appendicitis, acute gall bladder disease and symptomatic benign adnexal tumours in pregnancy.

The intended audience for this guideline is obstetricians and gynaecologists in secondary and tertiary care, general surgeons and anaesthetists. However, only laparoscopists who have adequate laparoscopic skills and who perform complex laparoscopic surgery regularly should undertake laparoscopy in pregnant women, since much of the evidence stems from specialised centres.

## 1. Purpose and scope

The scope of this guideline is the laparoscopic management of non-obstetric, abdominal conditions in pregnancy. Laparoscopy is widely utilised to diagnose and treat, acute and chronic, gynaecological and general surgical conditions. It is only in recent years that laparoscopy has become an acceptable surgical alternative to open surgery in pregnancy. To date there is little clinical guidance pertaining to laparoscopic surgery in pregnancy. This is why the British Society of Gynaecological Endoscopy (BSGE) commissioned this guideline using evidence grading as used by the Royal College of Obstetricians and Gynaecologists (RCOG) (appendix 1) with the help of contributors.

The conditions included in this guideline are acute appendicitis, acute gall bladder disease and symptomatic benign adnexal tumours in pregnancy. The intended audience for this guideline is obstetricians and gynaecologists in secondary and tertiary care, general surgeons and anaesthetists. However, only laparoscopists who have specialist laparoscopic skills and who perform complex laparoscopic surgery regularly should undertake laparoscopy in pregnant women.

## 2. Identification and assessment of evidence

MEDLINE, EMBASE, CINAHL and the Cochrane library were searched for relevant articles. Literature searches were performed in MEDLINE (from 1950 to September 2015), EMBASE (from 1980 to September 2015), CINAHL (from 1981 to September 2015) and the Cochrane library. No restrictions were placed on the searches in an attempt to reduce selection bias. The databases were searched using the relevant MeSH terms and keywords.

A subsequent update search ensured relevant papers were included up to February 2017.

The main search terms were ‘laparoscopy’ and ‘pregnancy’, these were used with combinations of the following words and appropriate synonyms, depending upon the area of laparoscopy in pregnancy being examined; anaesthesia, appendicectomy, cholecystectomy, ovarian cysts, performance and safety of imaging.

The results of the searches were systematically reviewed to identify relevant articles. The reference lists of selected papers were then searched to identify any additional articles not captured by the literature search. When a study, which was relevant to one of the other topic areas, was identified in a search, we cross-referenced to ensure that it was identified in the relevant search. Studies were included if they addressed the diagnosis and / or management of acute surgical conditions, such as acute appendicitis, acute cholecystitis and symptomatic benign adnexal tumours in pregnancy. Suspected ovarian malignancy, ectopic and heterotopic pregnancy were excluded (guidance regarding these conditions can be found in the National Institute of Clinical Excellence (NICE) Guideline CG122, 2011(NICE, 2011) and the NICE Guideline CG154, 2012 (NICE, 2012), respectively).

Where possible, recommendations were based on available evidence and the areas where evidence was lacking were annotated as ‘good practice points’. Further information about the assessment of evidence and the grading of recommendations may be found in appendix 1.

## 3. Introduction and background epidemiology

What is the incidence of non-obstetrical emergencies in pregnancy?

**The incidence of acute, non-obstetric abdominal surgical emergencies in pregnancy is low.**

C

**Clinicians should be aware of the possibility of appendicitis in pregnancy, but its incidence is lower than in the non-pregnant state.**

D

**Hormonal changes may increase the ultrasound scan findings of gallstones and sludge in pregnancy. However, the risk of gallbladder disease appears to be reduced.**

D

**The absolute numbers of torted and haemorrhagic ovarian cysts are low in pregnancy. Fertility treatment with GnRH analogues involving controlled ovarian stimulation appears to increase these risks.**

D

Non-obstetric abdominal surgery during pregnancy is rare and occurs in 1–2/1000 pregnancies (Silvestri et al., 2011; Balinskaite et al., 2017).

Evidence level: 2+

Two large cohort studies from the USA reported the incidence of appendicitis to be 1 in 1000 pregnancies (Abbasi et al. (2014) examined n=7037386 pregnancies, Mourad et al. (2000) n=66993 pregnancies (0.1%)). A Korean health registry study found that the prevalence of acute appendicitis in pregnant women (96 ± 97 per 100000) was significantly lower than that in non-pregnant women (206 ± 3 per 100000), (OR, 0.376; 95% CI, 0.31- 0.46, p <0.001) (Yuk et al., 2013).

The search identified one large series of 46075 pregnant women, that reported the incidence of biliary disease in pregnancy to be 0.16% (Swisher et al., 1994). In the general population the incidence is 10-15% with a female-to-male ratio of 4:1, despite the predilection for sludge and stone formation in pregnancy (Schirmer et al., 2005).

The reported incidence of ovarian cysts in pregnancy varies between studies, depending upon the study population, the inclusion criteria and the size of ovarian cysts. If a cut off of >3cm is used, the incidence in pregnancy is 1.2%, if a cut off of >5 cm is used the incidence in pregnancy is 0.05% (Duic et al., 2002; Zanetta et al., 2003; Katz et al., 2010). In non-pregnant women of reproductive age the incidence is 1.8%, if a cut off of > 4 cm is used (Borgfeldt and Andolf, 1999).

Ovarian torsion in pregnancy is rare in absolute terms. In Duic’s series, torsion, sub-acute torsion or haemorrhage necessitating a surgical intervention occurred in 1:4000 pregnancies. Katz et al. (2010) reported a torsion rate of 1:70000, with hospital admissions for pain required in 16% of women with cysts. The incidence of ovarian torsion in non-pregnant women is not known.

An association with ovarian stimulation for fertility treatment was observed in a retrospective review of 180 consecutive women of childbearing age over an 11-year period with surgically confirmed adnexal torsion; 48 women were pregnant (median gestational age 7 weeks), of those 39 women had received fertility treatment and nine had not. The relationship between ovarian enlargement and fertility treatment was not investigated (Tsafrir et al., 2012).

Evidence level: 3

## 4. Ovarian cyst accidents, appendicitis and cholecystitis causing complications specific to pregnancy

What are the specific concerns of ovarian cyst accidents, appendicitis and cholecystitis in pregnancy?

**Without appropriate surgical treatment, appendicitis and cholecystitis carry specific risks in pregnancy including generalised peritonitis and maternal sepsis resulting in miscarriage, preterm delivery and stillbirth. No data was identified regarding the effect of cyst accidents on pregnancy.**

D

When comparing pregnant to non-pregnant women, a large retrospective cohort study showed a higher rate of pre-operative systemic infection (PSI) and systemic inflammatory response syndrome (SIRS) in pregnant women (Silvestri et al., 2011). For appendicitis the incidence of PSI was 39.7% versus 33.6% (p<0.001) and for SIRS it was 35.7% versus 32.2% (p=0.001). For cholecystitis the incidence was 11.9% versus 5.2% (p=0.001) for PSI and 11.2% versus 4.8% (p=0.0010) for SIRS. The authors suggested that compounding factors might include suppressed maternal immunity in pregnancy, difficulty making the diagnosis and reluctance to operate. There was a higher rate of fetal loss and early delivery when appendicitis was complicated by generalised peritonitis or peritoneal abscess (p<0.05); 6% fetal loss and 11% early delivery in complex appendicitis versus 2% fetal loss and 4% early delivery in simple appendicitis (McGory et al., 2007).

No studies were identified that compared the incidence of ovarian cyst complications in pregnant and in non-pregnant women in our search. For instance, there are no data on whether cyst accidents lead to more severe peritonitis or haemorrhage when a woman is pregnant. The risk of torsion appears to decrease with increasing gestation in a retrospective series of 107 fertility patients (Zanetta et al., 2003). After one episode of torsion the recurrence risk of repeat torsion was 19.5% in pregnant women and 9.1% in non-pregnant women in a retrospective case control study (n=118) (Hasson et al., 2010).

Evidence level: 2-

Pregnancy complications secondary to ovarian cysts include fetal loss and premature delivery.

## 5. Safety of Laparoscopy

Is laparoscopy in pregnancy safe for mother and fetus?

**In comparison with open surgery, laparoscopy for adnexal and gallbladder disease has no increased risk for mother and fetus. Where appropriate surgical equipment and expertise is available, laparoscopy should be considered an appropriate surgical approach.**

B

**(Recommendations for appendicitis in pregnancy see 9.1)**

**There is no additional risk of fetal malformation or stillbirth in women who undergo non-obstetric surgery compared with pregnant women who do not undergo surgery. The maternal condition necessitating surgery may be associated with risk of miscarriage and preterm labour.**

C

**Only experienced laparoscopists should carry out these procedures and outcomes should be carefully monitored. Joint surgery between gynaecologists and surgeons is encouraged.**

✓

**The decision between laparoscopic and open routes of surgery should be based upon the available expertise, infrastructure, background history, gestation and the woman’s preference.**

✓

Previously pregnancy was thought to be a contraindication for laparoscopy, but now there are numerous publications, showing acceptable maternal and fetal outcomes. Most evidence is based on case series and systematic reviews of those.

Compared with laparotomy, laparoscopic surgery for ovarian cysts in pregnancy was associated with better maternal outcomes, and no increase in adverse obstetric outcomes (miscarriage, preterm delivery or fetal growth restriction) in a systematic review of 240 women (Liu et al., 2017).

Two systematic reviews of non-randomised controlled trial (RCT) studies of laparoscopy for gallbladder disease reported good maternal and fetal safety. Adverse fetal outcomes (OR=0.42, Confidence interval (CI)=0.28-0.63, p<0.001), maternal complications (OR=0.42, CI=0.33-0.53, p<0.001), or intra-operative immediate post-operative surgery related complications (OR=0.45, CI= 0.25-0.82, p=0.01) were all less frequent in laparoscopy than in laparotomy (Sedaghat et al., 2017). In a non-comparative systematic review, Nasioudis et al. (2016) reported the rate of fetal loss as 0.4%. Intraoperative maternal complications were 3.86% and postoperative maternal complications were 4%, of which fewer than 1/3 in each section could be classed as severe (see section 9.2). A systematic review of suspected appendicitis in pregnancy identified 11 non-randomised comparative studies of open (n=2816) and laparoscopic (n=599) treatment (Wilasrusmee et al., 2012). Fetal loss was significantly increased in the laparoscopy group (RR=1.91, CI=1.31-2.77).

Evidence level: 2++

However, the increased report of fetal loss was attributed to a single large study (McGory et al., 2007). No adverse fetal outcomes associated with laparoscopy were reported by the remaining smaller studies. McGory et al. (2007) studied retrospective administrative hospital records of pregnant (n=3133) and non-pregnant women (n=91656) who underwent open (n=73269) or laparoscopic appendicectomy (n= 24214). Gestational ages at surgery and at the time of fetal loss were not reported. Fetal loss was identified by diagnosis codes for miscarriage, dilatation and curettage or intrauterine death. Laparoscopy was associated with a higher rate of fetal loss (7%) compared with open appendicectomy (3%) (Odds ratio (OR)=2.31, CI=1.51-3.55, p<0.5) but early delivery was less common in laparoscopic appendicectomy (<1%) compared to open (8%) (p<0.5). In this study, it was not possible to make a causal link between laparoscopic appendicectomy and fetal loss. It has to be taken into account that the laparoscopic approach is the preferred approach in the first trimester, when spontaneous fetal loss occurs most frequently, and the association could be spurious.

When comparing laparoscopic to open appendectomy, Balinskaite et al. (2017) in a retrospective review of routinely collected hospital data in the UK reported higher incidence of spontaneous miscarriage (OR=2.36, CI=1.71 – 3.26). There was no increase in the other primary outcomes such as delivery by caesarean section, preterm delivery < 37 weeks’ gestation, low birth weight of <2500g, stillbirth, long inpatient stay and maternal death. They analysed data of the 6486280 pregnant women of whom 47628 (0.7%) underwent non-obstetric surgery, 26% underwent abdominal surgery, including 3061 appendicectomies. They reported that in the first trimester laparoscopic procedures were nearly 5 times more common than open surgery. Conversely, in the third trimester open procedures were 2.5 times more common than laparoscopic surgery. The authors stated that cause and effect could not be established and that the higher risk of miscarriage could be related to the preference of laparoscopic approach in the first trimester, when there is a higher chance of miscarriage.

Mazze et al. (1989) reviewed Swedish birth registries over a period of nine years, and investigated the risk of adverse fetal outcomes, after non-obstetric surgery in pregnancy. A total of 5405 operations (25% open abdominal - mainly appendicectomy, 19% gynaecological/urological and 16% laparoscopic) were reviewed. Out of 868 women who had laparoscopic surgery, 768 had surgery in the first, 29 in the second and 71 in the third trimester respectively. No direct comparison was made between an open and a laparoscopic approach. Mazze et al. (1989) concluded there was no additional risk of fetal malformation or stillbirth when compared with expected rates in non-surgical pregnant women. However, there was an increase in the risk of low birth weight (<1500 grams risk ratio 2.2, 95% CI 1.8-2.8; <2500 grams risk ratio 2.0, 95% CI 1.8-2.2); preterm birth <37/40 weeks (rate 7.5% versus 5.1% p=0.001) and neonatal death at 168 hours (risk ratio 2.1 CI 1.6-2.7) when compared with expected rates in non-surgical women. Neonatal deaths were associated with prematurity, but preterm births did not occur from delivery immediately after the operation but with an average delay of 21, 7 and 5 weeks in the first, second, and third trimester respectively. The relationship between the condition necessitating surgery in pregnancy and adverse outcomes of pregnancy was not investigated and could be a confounding factor.

Reedy et al. (1997) looked at 20-year period of health registry data (a proportion also included in Mazze et al. (1989) and compared open (n=1522) to laparoscopic (n=2181) surgery in pregnancy at gestations between four weeks and twenty weeks. When these women, who had undergone either open or laproscopic surgery, were compared to the normal pregnant population they had no increased risk of fetal malformations (open surgery versus total studied population risk ratio 1.08, 95% CI 0.85-1.11; laparoscopic surgery versus total studied population risk ratio 1.09, 95% CI 0.9-1.11).

No developmental or physical abnormalities were seen in children born to mothers who had laparoscopic surgery between 16 and 28 weeks’ gestation (including cholecystectomy, appendicectomy and surgery for small bowel obstruction) (Rizzo, 2003). The follow-up ranged from 1 to 8 years.

Evidence level: 2+

What are the maternal benefits to laparoscopic compared to open surgery?

**Laparoscopic surgery is associated with faster recovery, shorter hospital stay and a trend to lower rate of wound infection for pregnant women.**

C

Cox et al. (2016) using the US National Surgical Quality Improvement Program (NSQIP) which included 1999 pregnant women undergoing laparoscopic or open cholecystectomies or appendicectomies in non-perforated appendicitis, reported shorter operation time (p<0.0001), shorter hospital stay (2.3 ± 5.8 versus 3.3 ± 2.5, p<0.01), and fewer postoperative wound complications (0.67% versus 3.9%, p<0.01) for laparoscopic surgery.

Evidence level: 2+

In a retrospective review of 2000 cases of open and laparoscopic appendicectomies in pregnancy from the NSQIP database, laparoscopy was associated with fewer wound infections (p=0.04), return to theatre, other infections, respiratory morbidity, venous thromboembolism (VTE), and blood transfusion (p=0.048), whilst open cases had more pre-operative systemic infections (Erekson et al., 2012).

Wilasrusmee et al. (2012) conducted a meta-analysis of 11 non-RCT studies of pregnant women undergoing surgery for appendicectomy (n=599 laparoscopic, n=2816 open). Only three studies reported wound infection, which was not significantly reduced in laparoscopy (RR=0.91, CI=0.12 -7.18). Hospital stay was shorter by half a day (CI=1.76-0.78 days) with the laparoscopic approach.

Since the publication of Wilasrusmee et al. (2012) two comparative case series have been published. Laustsen et al. (2016) reported a small-scale comparison between 19 laparoscopic and 25 open appendectomies and reported fewer complications including wound infection; abscess, haematoma (5.3% versus 36%, p=0.03) and shorter hospital stay (2.6 versus 5.5 days, p = 0.004) with the laparoscopic approach.

Segev et al. (2016) compared 50 laparoscopic with 42 open appendicectomy cases in pregnancy. Two per cent of the open cases were conversions from laparoscopy. The laparoscopy group had a lower median gestational age at surgery (16 weeks versus 24 weeks, p<0.001), a shorter median hospital stay (5 days versus 3 days, p<0.001), and a lower rate of postoperative complications (8% versus 24%, p=0.04), with no difference in gestational age at delivery, Apgar scores, and rates of preterm delivery or fetal loss.

Evidence level: 2-

What are the maternal risks from laparoscopy in pregnancy?

**General laparoscopic surgical risks such as haemorrhage and herniation at the port site also apply to laparoscopy during pregnancy.**

A

**Due to enlargement of the uterus and subsequent limitations to visual field and surgical access there is an increased risk of vascular and organ trauma, in particular uterine perforation, although this risk has not been quantified.**

D

**Clinicians should counsel women about consequences of uterine perforation, which include subsequent uterine rupture, infections, preterm delivery, and laceration of the fetus or the placenta. The size of the perforation is likely to be of importance.**

✓

**Clinicians should be aware that there is increased risk of bleeding due to increased vascularity of uterus and adnexae, but this risk is currently not quantified.**

D

A systematic review on cholecystectomy in pregnancy (Nasioudes et al., 2016) gives a narrative account of non-pregnancy specific complications such as haemorrhage and herniation at the port site after laparoscopy. Another systematic review on cholecystectomy in pregnancy reports a maternal death associated with laparoscopic cholecystectomy in 767 cases (Sedaghat et al., 2017). Details of this death are missing despite contacting the authors.

Evidence level: 1-

There are only case reports on trauma to the uterus. Case series of less than five cases were not included in the guideline, with the exception of this section, where no other evidence was available. In one case the uterus was laparoscopically repaired after Veress needle and trocar injury. PROM occurred at 32 weeks. The child was well at 12 months (Joumblat et al., 2012). In a second case, inadvertent pneumo-amnion occurred at 21 weeks’ gestation during a negative laparoscopy, resulting in miscarriage (Friedman et al., 2002).

A case report details the rupture of uterine varicocele at laparoscopic appendectomy at 33 weeks, followed by conversion to emergency caesarean section, with good maternal and neonatal outcome (Holzer et al., 2011).

Evidence level: 3

## 6. Anaesthesia

No studies were identified which examined the maternal risks of anaesthesia for women undergoing laparoscopic surgery in pregnancy. Recommendations regarding the maternal anaesthetic risks for non-obstetric laparoscopic surgery in pregnancy have been extrapolated from both non-pregnant women having laparoscopy, and the delivery of anaesthesia in the pregnant population.

What are the maternal anaesthetic risks of laparoscopy in pregnancy and how can these specific risks be safely managed?

**The pre-operative anaesthetic review should include relevant features related to the pregnancy such as gestation and pregnancy related co-morbidities as well as the routine anaesthesia history and examination.**

✓

**There should be early involvement of an obstetric anaesthetist, or at the very least consultation with an obstetric anaesthetist.**

✓

**Aspiration prophylaxis should be administered, and a strategy for airway management should be made. General anaesthesia and endotracheal intubation are essential, and the use of a laryngeal mask airway is not recommended.**

D

**Creation of the pneumoperitoneum should be gradual, as should alterations in maternal positioning.**

✓

**Pregnant woman undergoing non-obstetric surgery are at an added risk for venous thromboembolism. Their risk for venous thromboembolism should be stratified and prophylaxis considered as per the Royal College of Obstetricians and Gynaecologists Green-Top Guidance.**

D

An anaesthetic assessment should include a thorough evaluation of the patient, including timely pre-operative resuscitation if necessary. Aspiration prophylaxis should be administered as part of the premedication, and an early strategy for airway management should be formulated. A rapid sequence induction should be considered to minimise the risk of pulmonary aspiration.

Establishment of pneumoperitoneum can be accompanied by marked changes in cardiovascular and respiratory physiology (Struthers and Cuschieri, 1998). Alterations in positioning and the creation of the pneumoperitoneum should be gradual, with vigilance and monitoring of the woman’s haemodynamic status.

Evidence level: 4

Non-obstetric surgery during pregnancy is associated with an increased risk of venous thromboembolism (VTE). As a minimum, the RCOG classifies any surgical procedure as an intermediate risk, whereby pharmacological prophylaxis with low molecular weight heparin should be considered (RCOG, Guideline No 37a and 37b, 2015).

Evidence level: 2-

What are the fetal risks associated with anaesthesia and what strategies can be employed to increase the safety for the fetus, when pregnant women require non-obstetric laparoscopic surgery?

**Modern anaesthetic agents, muscle relaxants and opioids are not thought to be teratogenic when used in therapeutic clinical doses and when the maternal physiology is maintained.**

✓

**Clinicians should ensure the utero-placental blood flow is maintained by avoiding maternal hypotension.**

D

**Maternal arterial CO_2_ should be controlled, avoiding hypo- and hypercapnia, to maintain optimal utero-placental flow and thus avoid fetal acidosis.**

D

**End-tidal CO_2_ (ETCO_2_) can be used as a surrogate marker for arterial CO_2_.**

D

No primary studies were identified that selectively investigated the fetal anaesthetic risks of laparoscopic surgery separately from the risks of the surgical intervention. There are no primary studies that distinguish the effects of anaesthetic factors from surgical factors with regard to fetal risks.

The risks of anaesthesia for the fetus of pregnant women undergoing non-obstetric laparoscopic surgery can broadly be divided into two. Firstly, the risks related to pharmacological agents used in anaesthesia and the risk of teratogenicity. Secondly, the risks related to a reduction in uteroplacental blood flow, secondary to changes in maternal mean arterial pressure, partial pressure of arterial carbon dioxide (PaCO_2_), oxygenation, aorto-caval compression and increased intra-abdominal pressure.

No anaesthetic agents have yet been proven to be teratogenic in humans when used in clinical doses and when normal physiology is maintained (Kuczkowski, 2006).

Evidence level: 4

Utero-placental blood flow is essential for oxygen delivery to the fetus and impairment to this can threaten fetal viability (Chestnut et al., 2009). When maternal blood pressure is maintained close to baseline and end-tidal CO_2_ controlled between 3.7-4.3 kPa, no adverse outcomes were reported (Steinbrook et al., 1996; Bhavani-Shankar et al., 2000; Rajmohan et al., 2013).

ETCO_2_ can be used as a surrogate marker for maternal arterial CO_2_ monitoring, and invasive arterial monitoring is not routinely required in otherwise healthy and stable women, however the decision should be made on a case by case basis (Bhavani-Shankar et al., 2000).

Evidence level: 3

Uterine displacement can improve cardiac output and uteroplacental flow and should be used unless it adversely impacts on the ability to carry out the surgery in a timely and effective manner, when gestational age is greater than 18 weeks (Cluver et al., 2013).

Evidence level: 1++

What type of anaesthesia should be used?

**In most cases general anaesthesia should be employed.**

✓

There is limited evidence on the use of regional anaesthesia for laparoscopy in pregnancy. The benefits of general anaesthesia include securing the airway to reduce the risk of aspiration, good muscle relaxation to allow excellent surgical conditions and controlled ventilation to regulate maternal PaCO_2_. In addition, general anaesthesia can avoid any discomfort that an awake woman may endure, related to either a high neuraxial sensory block level for an adequate pneumoperitoneum, or steep positioning.

## 7. Peri- and intra-operative laparoscopic management of non-obstetric emergencies

Who should be involved in the management of pregnant women requiring laparoscopy for abdominal non-obstetric conditions?

**A multi-disciplinary team should be in charge of the care of pregnant women requiring laparoscopy. Depending on the individual case this team may include gynaecologists, general surgeons, anaesthetists, obstetricians and neonatologists.**

✓

**Laparoscopic surgery in pregnancy should be performed by advanced laparoscopic surgeons with appropriate training and competencies in order to reduce complications and operating times.**

D

**Pregnancy should not be a reason to delay urgent surgery.**

✓

**If the expertise to undertake laparoscopic approach in pregnancy is lacking, then the open route is acceptable since maternal and fetal outcomes are good.**

✓

**Both laparoscopic and open routes are acceptable, depending on circumstances, since maternal and fetal outcomes are equally acceptable.**

✓

General surgeons with a large caseload (top quartile of participating surgeons in annual number of cholecystectomies) experienced fewer maternal (1% versus 14%, p<0.0001), fetal (4% versus 10%, p<0.0001) and surgical (10% versus 13%, p<0.05) complications than other surgeons. Their patients also had a shorter length of stay (4 versus 5 days, p<0.0001) in a retrospective cohort study of 9714 predominantly laparoscopic surgeries (89% cholecystectomies) in pregnancy (p<0.0001) (Kuy et al., 2009).

The maternal benefits of laparoscopy (shorter operating time and hospital stay, minor complications) were not observed in cases of perforation of appendix or gallbladder (Cox et al., 2016). Timely surgical intervention is important in order avoid the risk of perforation and subsequent sepsis. Higher rates of perforation were observed when surgery was delayed for more than 24 hours after onset of symptoms (Tamir et al., 1990).

Maternal and fetal outcomes are satisfactory in both open and laparoscopic interventions (Aylin et al., 2016), therefore open surgery in experienced hands is preferable to delayed laparoscopic treatment.

Evidence level: 2-

At what gestation should laparoscopic surgery in pregnancy be performed?

**When deciding on the route of surgery clinicians should be aware that recent small series have shown good maternal and fetal outcomes for laparoscopic appendicectomy, cholecystectomy and adnexal surgery up to 34 weeks gestation, which extends historical recommendation to limit laparoscopic surgery to the second trimester.**

D

**Any surgery in pregnancy is associated with maternal and fetal risks. Non-urgent surgery should be postponed until after pregnancy.**

✓

According to previous American College of Obstetrics and Gynecology (ACOG) committee opinion, the second trimester is the best time to carry out non-urgent laparoscopic surgery, because preterm contractions and miscarriage are least likely (Committee Opinion No. 696, 2017). Furthermore, access to pelvic organs and the gallbladder is easier because the uterus is smaller than in the third trimester. However, there is emerging evidence showing good maternal and fetal outcomes outside the second trimester.

Evidence level: 4

A series of third trimester cases managed laparoscopically included five cholecystectomies, four appendicectomies, and two adnexal operations (Upadhyay et al., 2007). One patient went into labour at 34 weeks following appendicectomy complicated by peritonitis. Another patient (29 weeks) was converted to open salpingo-oophorectomy for torsion after diagnostic laparoscopy due to operator preference but required emergency laparotomy and caesarean section due to bleeding from the ovarian pedicle. The two complications are not likely to be due to the laparoscopic approach.

Evidence level: 3

Another observational study compared 117 laparoscopic operations (adnexal torsion, persistent cysts, cholecystitis and appendicitis) in the first trimester (n=71, mean gestational age 7.7 ± 1.9 weeks) with laparoscopic surgeries in the second and third trimesters (n= 46, mean gestational age 18.1 ± 4.3 weeks, 11 cases in third trimester, up to 34 weeks) (Weiner et al., 2015). No difference was found between the two groups regarding surgical complications and pregnancy outcomes. In both groups half of the deliveries were before 37 weeks.

Evidence level: 2-

Where should laparoscopic surgery in pregnancy be performed?

**Laparoscopic surgery in pregnancy should be carried out in settings where adequate time, laparoscopic expertise and monitoring facilities are available.**

✓

**After the age of fetal viability pregnant women undergoing laparoscopic surgery should be treated in a unit with adequate obstetric and neonatal facilities in case the immediate delivery of the baby is indicated.**

✓

The studies which informed this guideline originated mainly from tertiary care units in developed countries and so the recommendations cannot be extrapolated to all settings.

What interventions are needed when planning laparoscopic surgery in pregnancy?

**Fetal heart Doppler ultrasound monitoring may be done before and after surgery to confirm fetal wellbeing and reassure the mother. There is no need for routine intraoperative monitoring.**

D

**If there is a risk of pre-term delivery antenatal corticosteroids for fetal lung maturation and magnesium sulphate for fetal neuro-protection should be administered dependent upon the gestation of the fetus.**

A

**Anti-D administration is not deemed necessary according to guidelines since aparoscopic surgery is not included in the list of potentially sensitising events.**

A

**Routine tocolysis for women undergoing laparoscopic or open surgery in pregnancy is not recommended because it has not been shown to improve outcomes.**

B

**Caution needs to be exercised in steroid administration in maternal sepsis.**

✓

In the past intraoperative fetal heart monitoring, especially with open surgery, was seen as mandatory. Newer large case series have shown good outcomes without routine intra-operative fetal heart monitoring during laparoscopy (Kirshtein et al., 2009; Chung et al., 2013).

Evidence level: 3

A small quasi-experimental study showed no Doppler anomalies of the fetal heart or the maternal uterine arteries (measured trans-vaginally) during laparoscopic surgery on ovarian cysts using a routine laparoscopic approach with less than 12 mmHg pneumoperitoneum (Candiani et al., 2012).

Evidence level: 2+

Intra-operative monitoring may be required only in selected cases and when emergency delivery of the fetus is being considered. According to ACOG the following items need to be present before considering fetal monitoring: fetal viability, technical feasibility for intraoperative electronic fetal monitoring, an obstetrician willing to intervene for fetal indications, maternal consent for Caesarean section (CS) (desirable) and feasibility to interrupt laparoscopic surgery for emergency CS (Committee Opinion No. 696, 2017). Intraoperative monitoring during laparoscopy can be achieved by trans-vaginal or trans-abdominal ultrasound scanning with a steep left tilt to overcome the pneumoperitoneum.

If there is a risk of preterm delivery of a viable fetus, antenatal corticosteroids between 24±0 and 35±6 weeks and magnesium for fetal neuro protection should be used up to 33±6 in accordance with existing NICE guidance (NICE, 2015). Urgent surgery should not be delayed for administrating corticosteroids.

A systematic review showed no difference in the preterm delivery rate between women who received prophylactic tocolysis and those who did not (Walsh et al., 2008).

Laparoscopic surgery is generally not considered a sensitising event and therefore routine administration of prophylactic anti D is not required (Qureshi et al., 2014).

Evidence level: 2++

## 8. Intraoperative considerations

The following section discusses intraoperative issues relating to laparoscopy in pregnancy. Specific conditions (appendectomy, gallbladder disease and adnexal surgery) will be discussed in section 9.

**Clinicians would be aware that intrauterine manipulation is contraindicated in pregnancy. Hence collaboration with the anaesthetic team (positioning, tilting) and experienced operating assistance is required.**

✓

In order to improve surgical access without an intrauterine manipulator, alternative strategies include digital vaginal manipulation, planning the placement of surgical ports according to pathology and uterine size, tilting of the surgical table and consideration of thirty-degree laparoscope. Bearing in mind that uterine surface in pregnancy is more friable and can bleed more easily on contact even with blunt instruments, a ‘no touch’ approach should be adopted.

## 8.1 Ports

How should the primary and secondary ports be placed?

For generic entry techniques refer to the RCOG recommendations preventing entry-related gynaecological laparoscopic injuries for non-pregnant women (Green-top Guideline No 49, 2008). This section will address the issues specifically relevant to laparoscopy in pregnancy.

Where should the primary port be placed in pregnancy?

**The location of the primary port will depend on the level of the uterine fundus.**

D

**The uterine size should be determined by palpation or ultrasound.**

✓

**In the absence of RCTs clinicians should choose their primary port location including umbilical, supra-umbilical / sub-xiphoid and Palmers’ point (left upper quadrant in the mid- clavicular line) according to uterine size, location of pathology and operator experience.**

D

**It has been suggested that in the late second and the third trimesters primary port sites could include 1-2cm below costal margin in the left (Palmers’ point) or right mid-clavicular line or 3-6 cm above the umbilicus in the midline.**

D

**Insertion of an orogastric tube for gastric decompression maybe helpful when Palmer’s point is used for access.**

✓

No study was identified that randomised for location of the primary port, thus no recommendations can be made for one location over another.

Researchers describe a variety of different locations for primary ports depending on gestational age (Lyass et al, 2001; Lenglet et al., 2006; Machado et al., 2009; Bani Hani, 2007; Kirshtein et al., 2009; Balthazar et al., 2011; Jeong et al., 2011; Wilasrusmee et al., 2012; Koo et al., 2012; Peng et al., 2013; Chen et al., 2014; Nasioudis et al., 2016; Sedaghat et al., 2017).

In order to avoid uterine perforation and restricting views from having the camera port too close to the uterus, it is recommended to adjust the port location according to the fundus (Jeong et al., 2011). Therefore, the fundus should be palpated before insufflation. In very obese women transabdominal ultrasound and obstetric guidance may be required. Upadhyay et al. (2007) reported 11 laparoscopies between 26-28 weeks’ gestation (appendicectomy, cholecystectomy and adnexal surgery) using 1-2 cm below the costal margin on the left or right mid-clavicular line using Veress’ insufflation in 10 cases and Hasson’s in one case with no access-related complications.

Evidence level: 3

Should the Veress or Hasson technique be used for primary port placement?

**The Hasson technique has been reported inside or above the umbilicus in the midline.**

D

**With either approach clinicians should be mindful of the possibility of uterine injury.**

D

**The benefits of the Hasson technique may include reducing the risk of uterine trauma and spillage of contents of ovarian cysts.**

✓

**Clinicians should be aware that in experienced hands direct (gasless) entry may be an alternative, but there is insufficient information in late pregnancy to suggest this as a routine approach.**

✓

Although there are no studies randomised for Hasson’s and Veress’ entry techniques, a number of authors have recorded case series using these approaches throughout all gestations for cholecystectomy (Bani Hani, 2007), appendicectomy (Lyass et al., 2001), and adnexal surgery without entry-related complications (Balthazar et al., 2011). The Hasson technique has been reported inside or above the umbilicus in the midline.

Chen et al. (2014) reported direct entry in 33 laparoscopies for ovarian cysts in second trimester (mean gestation 16.8) without entry-related complications. The direct entry technique was described as placing an optical trocar under visual control in the umbilicus, whilst elevating the umbilicus with a towel forceps. Park et al. (2010) described direct entry via Palmer’s point (left upper quadrant in the mid-clavicular line) in n=8 cases of pregnancy appendicectomies (2 in first trimester, 5 in second trimester, one in third trimester) using a 5 mm trocar. No maternal of fetal complications were observed. The incidence of port entry complications is very low, and the studies above are too small to detect entry related complications.

Evidence level: 3

Where should secondary ports be placed?

**Secondary port placement will be dictated by uterine size, pathology and operative approach.**

D

**Pre-surgical planning is paramount due to the challenges of accessing the pathology, given the limited degrees of freedom in laparoscopic surgery and the added obstacle of the size of the pregnant uterus. Ipsilateral port placement may circumvent this obstacle.**

✓

A systematic search returned no randomised control trials, but authors of case series of appendicectomy (Kirshtein et al., 2009; Jeong et al., 2011; Miloudi et al., 2012), and cholecystectomy in pregnancy stated that secondary port placement is dictated by uterine size and pathology (Sungler et al., 2014).

Evidence level: 3

Secondary port placement should be considered on the same side (ipsilateral port placement) as the identified pathology as this technique prevents the surgeon from having to instrument across the pregnant uterus.

Evidence level: 4

## 8.2 Pneumoperitoneum in pregnancy

What insufflation pressures and operative pressures should be used in laparoscopic surgery in pregnancy?

**An intraabdominal pressure of 20-25 mmHg should be used for gas insufflation before inserting the primary trocar .**

✓

**Clinicians should be aware that current evidence supports operating pressures of 12 mmHg.**

D

Current recommendations on operating pressures in pregnancy are in keeping with recommendations for the non-pregnant state. There are no studies on insufflation pressure during port insertions in pregnancy. The recommendation for an insufflation pressure of 20-25 mmHg is extrapolated from the RCOG recommendation in non-pregnant women (RCOG, Green-top Guideline No 49, 2008). Since these pressures are only maintained for a short duration until the primary port is placed, they are unlikely to harm the fetus.

Evidence level: 4

No adverse changes to feto-maternal perfusion or adverse pregnancy events were recorded at less than 12 mmHg pneumoperitoneum in a quasi- experimental setting using intraoperative Doppler studies (Candiani et al., 2012). This was backed up by numerous case reports showing no adverse effects on the fetus at operating pressures at or below 12 mmHg (Lyass et al., 2001; Mathevet et al., 2003; Yuen et al., 2004; Lenglet et al., 2006; Kirshtein et al., 2009; Jeong et al., 2011; Koo et al., 2012; Peng et al., 2013; Chung et al., 2013; Chen et al., 2014; Minig et al., 2016).

Evidence level: 3

## 8.3 Choice of laparoscope

What diameter laparoscope is preferred for laparoscopic procedures during pregnancy?

**Both 5 and 10 mm diameter laparoscopes have been used in pregnancy and choice of diameter depends upon the surgical requirements and availability of equipment.**

✓

The benefits of using a 10 mm diameter laparoscope include better quality of image and the possibility of removing a larger specimen through the camera port. The benefits of a smaller 5 mm laparoscope include the need for smaller incisions with better cosmesis and the ability to insert them through secondary ports to gain different views. Although numerous authors describe their use of 5 and 10 mm laparoscope, no data specifically comparing laparoscope diameter during pregnancy were identified.

Evidence level: 4

What degree of laparoscope is preferred for laparoscopic procedures during pregnancy?

**The choice of degree of laparoscope depends upon the preference of the surgeon and the surgical situation. Skilful use of a 30-degree laparoscope might improve the visual field in the presence of a large uterus.**

✓

There are no studies evaluating the optimal degree of laparoscope that should be used in pregnancy.

Evidence level: 4

Traditionally, many gynaecologists use 0 degree and general surgeons more often use 30 degree laparoscopes. However, extrapolating from non-pregnant laparoscopic surgery on large fibroid uteri, a 30 degree scope can improve visibility in the pelvis in trained hands.

Evidence level: 3

What retrieval technique should be used to remove surgical specimens during laparoscopic surgery in pregnancy?

**The choice of extraction method for surgical specimens during laparoscopic surgery in pregnancy should be in accordance with the preference of the operating surgeon.**

D

**Infected and potentially dangerous specimens should be contained in a tissue bag.**

✓

Consideration should be given to the use of a tissue bag to avoid peritoneal spill of cystic contents, bearing in mind the likely preoperative diagnosis. For example, care should be taken not to spill contents of dermoid cysts to avoid chemical peritonitis and spillage of potentially malignant cysts as clinical assessment cannot absolutely preclude malignancy (RCOG, Green-top Guideline No 62, 2011).

A systematic search identified 10 case series that mention techniques of tissue removal at laparoscopy during pregnancy between 5-34 weeks’ gestation (Lee et al., 2004; RCOG, Green-top Guideline No 49, 2008; Kirshtein et al., 2009; Machado and Machado, 2009; Koo et al., 2011a; Chung et al.,2013; Scheib et al., 2013; Chen et al., 2014; Minig et al., 2016). The indications for surgery included appendicectomy, cholecystectomy and adnexal surgery. The devices used included endobag, endocatch, endopouch and sterile condoms.

Evidence level: 3

Bearing in mind the likely diagnosis of the specimen, consideration should be given to carrying out the dissection within the bag as well as using it for removal.

No comparative data to guide preferred techniques for tissue extraction specimen were identified. There was no data on power morcellation, which should be discouraged in pregnancy due to the risk of uterine trauma.

What energy modalities should be used during laparoscopic surgery in pregnancy?

**Ultrasound, bipolar and monopolar energy sources are safe to use during laparoscopy in pregnancy.**

D

**The operating surgeon should choose energy modality based on his or her own preference. The surgeon should be mindful of the principles of safe electrosurgery in laparoscopy.**

✓

No papers were available that investigated energy use during laparoscopic procedures in pregnant women as their main research question. There is no evidence that electrosurgery in a pregnant woman is harmful to the fetus or embryo. When monopolar energy is used it is recommended that the return plate should not be placed such that the uterus is between the electrode and the plate.

Amniotic fluid, which is electrolyte rich, protects the fetus from concentration of current and there is no neuromuscular stimulation at the output frequency of electrosurgical generators. Whilst there is a discrepancy between advice from different manufacturers, published literature does not suggest increased risk of energy related complications with any type of energy devices including monopolar during pregnancy.

Nine case series’ reported on the use of energy modalities but this was not the main topic under investigation (Mathevet et al., 2003; Kirshtein et al., 2009; Park et al., 2010; Lee et al., 2010; Jeong et al., 2011; Chung et al., 2013; Peng et al., 2013; Chen et al., 2014; Takeda et al., 2014). The range of energy sources, which were used without complications, include ultrasonic, bipolar, and monopolar energy. Monopolar energy was reported as being used by Lee et al. in 2010. Lee et al. (2004) used monopolar scissors during 29 ovarian cystectomies between 6-16 weeks’ gestation without any operative complications. Mathevet et al. (2003) also used monopolar scissors in addition to bipolar diathermy in 48 pregnant women undergoing laparoscopic adnexal surgery (17 in first trimester, 27 in second and 4 in the third trimester). There were no intraoperative complications but one woman suffered a miscarriage at 17 weeks three days after surgery, which was not ascribed to the operating technique after review of the operative video. The use of Harmonic scalpel ® reported in 2 case series (Chung et al., 2013; Park et al., 2010). Chung et al. (2013) carried out 22 laparoscopic appendicectomies (6 in first, 13 in second and 3 in third trimester). No intra-operative complications occurred. Park et al. (2010) reported the use of Harmonic scalpel ® in 8 cases of appendicectomy during pregnancy (2 in first trimester, 5 in second trimester, one in third trimester) with no operative complications.

Evidence level: 3

Given the restricted access and visibility in laparoscopy in pregnancy the surgeon needs to respect electrosurgical principles to avoid trauma. General safety rules for monopolar diathermy apply (avoiding indirect thermal damage, pedicle effect, avoid coupling, checking for faulty insulation).

## 8.4 Closure Techniques

What wound closure techniques are recommended in pregnancy?

**The risk of hernia formation is 1-2% in incisions greater than 10 mm, therefore the fascia should be closed.**

D

No data were identified for port closure after laparoscopy in pregnancy. In non-pregnant women ports greater than 10 mm should be closed by formal sheath closure with a port closure system, such as Endoclose ® (with pneumoperitoneum maintained) or by using a J-needle, unless the Hasson entry technique has been used, in which case previously placed stay sutures are tied together. The risk of postoperative hernia formation is greater at the lateral port sites, especially taking into account the impact of the enlarging uterus on the abdominal wall stretch, which may further increase the risk of herniation. The skin can be closed by a variety of techniques e.g. subcuticular polyglactin and skin glue.

Evidence level: 4

## 8.5 Use of drains

Should an abdominal drain be inserted peri-operatively on completion of laparoscopic procedures in pregnancy?

**The operating surgeon should decide whether it is necessary to use a drain based on their preference and assessment of the individual case.**

D

Drain placement has been only described in the context of laparoscopic appendicitis in pregnancy, not for cholecystectomy or adnexal surgery. Comparative data between routine placement (Park et al., 2010; Chung et al., 2013), and selective placement (Jeong et al., 2011), is lacking.

Evidence level: 3

## 8.6 Post-operative care

What maternal care and fetal monitoring should be offered post-operatively?

The literature search did not return any studies that investigated fetal monitoring after surgery. Fetal heart Doppler ultrasound monitoring may be done after surgery to confirm fetal wellbeing and reassure the mother.

**Antibiotics should be used if there is an infective process. The choice of antibiotic should be based upon local anti-microbial guidance and drug safety in pregnancy.**

✓

**In the case of elective surgery for adnexal masses, antibiotics would not be routinely required.**

D

**Good analgesia, adequate rehydration to maintain euvolaemia and measures to prevent postoperative nausea and vomiting should be integrated into maternal postoperative care.**

✓

In a prospective case series of adnexal cysts operated on electively in the first trimester in 12 women no routine antibiotics were given and there were no complications including infections (Minig et al., 2016).

Evidence level: 3

## 9. Management of the commonest laparoscopically treatable abdominal emergencies in pregnancy

### 9.1 Appendicitis

Is there a role for expectant management of appendicitis in pregnancy?

**In suspected appendicitis in pregnancy timely surgical intervention is preferable as delay may be associated with adverse maternal and fetal outcomes.**

C

Two large retrospective studies were identified comparing conservative to operative approach for suspected appendicitis in pregnancy. Abbasi et al. (2014) reported a higher rate of complications in women who had antibiotics only compared to open or laparoscopic appendicectomy. This study also showed higher rates of septic shock (OR=6.3, CI=1.9-20.8), peritonitis (OR=1.6, CI=1.3-2.1) and increase in venous thromboembolic disease (OR=2.5, CI=0.9-7.4), when comparing pregnant women who underwent treatment with antibiotics (n=412) with those who underwent open (n=3421) or laparoscopic surgery (n=3279).

Cheng et al. (2015) investigated outcomes in pregnant women with appendicitis and antibiotic conservative treatment (n=78), open (n=653) and laparoscopic (n=128) surgery and compared outcomes with pregnant women without appendicitis (n=3436) from a national database. Pregnant women who had conservative treatment for appendicitis had a higher incidence of preterm labour (OR=2.47, CI=1.17-5.24) and pregnancy loss (OR=31.37, CI=13.12-75.01) than pregnant women who did not have appendicitis. Compared to pregnant women without appendicitis, women with open appendicectomy had significantly increased rates of preterm labour (OR=2.76, CI=2.06-3.70), pregnancy loss (OR=14.34, CI=7.70-26.71) and caesarean delivery (OR=1.24, CI=1.05-1.48). In contrast, women who underwent laparoscopic surgery had no statistically increased rates of preterm labour (OR=1.26, CI=0.57- 2.73), or caesarean delivery (OR=1.31, CI=0.91-1.88) compared to women without appendicitis. The risk of miscarriage was increased in all women who had appendicitis in pregnancy compared to pregnant women without appendicitis with conservative treatment 11.5% (OR=31.37, CI=13.12-75.01), open appendicectomy 5.7% (OR=14.34, CI=7.70- 26.71), laparoscopic appendicectomy 5.5% (OR=13.88, CI=5.50-35.04). There was no direct comparison between the antibiotic and the surgical groups. All three outcomes were similar when the two surgical approaches were compared.

Evidence level: 2+

Tamir et al. (1990) reported that a 24-hour delay in operating (laparotomy) in suspected appendicitis in pregnant women led to a 66% increase in perforation. Retrospectively, laparotomy occurred within 24 hours of symptom onset in 19/54 (35%) cases. Perforation was seen in 23/54 (42%) women, all of whom had symptoms exceeding 24h (p<0.0005).

Should the laparoscopic approach to appendicectomy be preferred over laparotomy in pregnancy?

**Whilst maternal outcomes are good, controversy exists regarding the association of laparoscopic appendicectomy and miscarriage. Further research is needed to distinguish between association and causality. In view of this, it is not possible to recommend one approach over the other and only experienced laparoscopists should carry out these procedures and outcomes should be monitored.**

C

A systematic review of 11 non-randomised comparative studies of open (n=2816) and laparoscopic (n=599) cases for suspected appendicitis in pregnancy was identified (Wilasrusmee et al., 2012). Most cases were from the second trimester, but cases from all trimesters were included. There was no data on complexity of cases in either group. Fetal loss was significantly increased in the laparoscopy group (RR=1.91, CI=1.31-2.77).

Evidence level: 2++

The adverse findings reported by Wilasrusmee et al. (2012) were influenced by a single study by McGory et al. (2007) without which there would be no increase in fetal loss associated with laparoscopy. McGory et al. (2007) studied retrospective administrative hospital records of pregnant (n=3133) and non-pregnant women (n=91656) who underwent open (n=73269) or laparoscopic appendicectomy (n= 24214) and reported a higher rate of fetal loss compared with open appendicectomy (OR=2.31, CI=1.51-3.55).

Evidence level: 2-

A small retrospective study was published after the systematic review by Wilasrusmee et al. (2012). Laustsen et al. (2016) compared 19 laparoscopic with 25 open appendicetomies and reported no miscarriages, no differences in Apgar score, weight, length and gestational age birth and improved maternal outcomes.

A large retrospective UK study on hospital data included 3061 appendicectomies (Aylin et al., 2016; Balinskaite et al., 2017). Laparoscopic appendicectomies were associated with an increased risk of spontaneous miscarriage (RR=2.36, CI=1.71-4.41), but very few (1.8%) occurred during immediately after laparoscopic appendicectomy. No differences were observed in risk of in preterm delivery <37/40, maternal death, long inpatient stay and low birth weight <2500 g. They reported that in the first trimester laparoscopic procedures were nearly 5 times more common than open ones. Conversely, in the third trimester open procedures were 2.5 times more common than laparoscopic ones.

Both studies have a large risk of a systematic distortion in measuring the true frequency of miscarriage due to laparoscopy because of the over-representation of first trimester pregnancies in the laparoscopy group.

Evidence level: 2+

Hence the risk of spontaneous miscarriage associated with appendicectomy during pregnancy should be interpreted with caution.

### 9.2 Gallbladder disease (Symptomatic gallstones and acute cholecystitis)

Is there a role for expectant management of cholecystitis in pregnancy?

**A conservative approach to gallbladder disease (symptomatic gallstones and acute cholecystitis) in pregnancy is associated with higher maternal morbidity than surgery.**

D

**Clinicians should be vigilant about complications of gallbladder disease such as gallstone pancreatitis, since this may be associated with a high risk of fetal mortality.**

D

**In pregnant women with biliary colic, supportive care will lead to resolution of symptoms in most cases. Complicated gallstone disease requires a more proactive approach.**

✓

The search identified one systematic review of moderate quality (Date et al., 2008), and a retrospective case series (Othman et al., 2012), that compared conservative, endoscopic retrograde cholangiopancreatography) and laparoscopic management. 112 pregnant women (first 29, second 43 and third trimester 40) were analysed retrospectively according to their approach to treatment. In the first and third trimester there were more conservative than active (laparoscopic and ERCP) treatments, but outcomes were not reported by gestation. Conservative treatment was associated with more recurrent biliary symptoms (30/50 versus 4/31, p=0.0002) mean visits to the emergency department (1.7 versus 1.1, p=0.0006) and mean days spent in hospital (1.5 versus 1.2, p=0.034) and caesarean section delivery (15/43 versus 2/25, p=0.04), but the duration of hospital stay (5 days versus 6.5 days, p=0.07) and the mean fetal birth weight (2752 g versus 2999 g, p=0.1) were not significantly different.

Date et al. (2008) systematically reviewed six case series, comparing conservative with surgical management of cholecystitis (open and laparoscopic) and showed no significant difference in the incidence of preterm delivery (3.5% versus. 6.0%, p=0.33) or fetal mortality (2.2% versus 1.2%, p=0.57) (Caspi et al., 2000). There was no case of maternal or fetal mortality in 20 reports of laparoscopic cholecystectomy and 9 reports of ERCP. In 12 reports of gallstone pancreatitis, fetal mortality was 6/75 versus 1/38 (p=0.28) in conservative and surgical groups respectively.

Evidence level: 2-

Should the laparoscopic approach to cholecystectomy be preferred over laparotomy in pregnancy?

**Laparoscopic cholecystectomy appears to be associated with better composite maternal and fetal outcomes than open approach, fewer surgical complications and shorter hospital stay.**

C

The search identified a systematic review comparing laparoscopic and open cholecystectomy in pregnancy (Sedaghat et al., 2017). Sedaghat et al. (2017) reviewed eleven studies including 10632 pregnant women with gallbladder disease or symptomatic gallstones who underwent open or laparoscopic cholecystectomy. All studies were retrospective, comparative and non-randomised. Seven studies reported the patients’ trimester at the time of surgery (161 cases). The first and second trimester predominated (first trimester 44/161, second trimester 102/161, third trimester 15/161) but outcomes were not stratified by trimester. One maternal death was reported in connection with laparoscopic cholecystectomy at 20 weeks for chronic cholecystitis 2 weeks postoperatively due to intra-abdominal haemorrhage from a non-identified source. Composite fetal complications (OR=0.42, CI=0.28-0.63, p<0.001), composite maternal complications (OR=0.42, CI=0.33-0.53, p<0.001), and composite surgical complications (OR=0.45, CI=0.25-0.82, p<0.01) were all less frequent in the laparoscopy group. There was no significant difference in fetal mortality, (OR=0.39, CI=0.07-2.19, p=0.29), or preterm delivery before 27/40 (OR=1.35, CI=0.41-5.14, p=0.59) between the open and laparoscopic group. Operation time was not significantly different in four studies that reported it (86.2 min versus 85.9 min, p=0.98). Length of hospital stay was reported in 5/11 studies and was significantly shorter in the laparoscopic group (mean 3.2 versus 6.0 days p=0.02).

Evidence level: 2++

### 9.3 Ovarian cysts and masses

Should surgery for ovarian cysts be preferred over the conservative approach in pregnancy?

**Women with asymptomatic simple cysts may be managed conservatively in pregnancy provided that symptoms are absent or acceptable to the woman.**

D

**Women with large, non-torted symptomatic cysts who wish to avoid surgery may be offered aspiration under ultrasound guidance during pregnancy, with definitive cystectomy after delivery if required.**

D

**The risk of torsion of ovarian cysts requiring emergency surgery in pregnancy is low and, in most cases, surgery may be delayed until the woman becomes symptomatic, with good fetal outcomes.**

D

A systematic search identified a retrospective series that observed the rate of torsion and malignancy by adnexal mass size in 470 women who underwent surgery for adnexal masses during all trimesters of pregnancy between 2002-2009 (Koo et al., 2011b). Fifty-five women (11.7%) had torsion and 28 women (31.8%) had emergency surgery for this indication. Torsion was more likely to occur when cysts were between 6-10cm than if they were smaller or larger than that (OR=2.68, CI=1.35-5.40, p<0.006). More than half of the torsions occurred in the first trimester and corpus luteum was the most common cyst type. Risk of torsion was not directly proportional to the cyst size.

Evidence level: 2-

A systematic search identified four retrospective and two prospective case series that evaluated conservative management of ovarian cysts in pregnancy (Condous et al., 2003). No evidence was found that recommended conservative treatment when an acute cyst accident was suspected.

Condous et al. (2004) followed up one hundred and sixty-one women with 166 ovarian cysts diagnosed by first trimester ultrasound (43% asymptomatic, 56.3% had pain or vaginal bleeding) throughout pregnancy with serial scans every 4-6 weeks until either the cyst resolved or intervention was required. Expectant management of ovarian cysts in pregnancy appeared to be safe with a low intervention rate of 4.2% (one evacuation of retained products of conception (ERPC) and laparoscopic cystectomy, three cystectomies at term section, two laparotomies in second trimester, aspiration in second trimester), 3% underwent torsion but only 0.13% of women required emergency surgery (laparotomy in second trimester, cystectomy at term section and second trimester cyst aspiration).

Zanetta et al. (2003) followed up 72 women with ovarian cysts in pregnancy greater than 3cm (after excluding those who were scanned in acute pain and required emergent surgery for torsions at presentation). Only two women required intervention (one had a cystectomy at caesarean section at 37 weeks for torsion, and one trans-cutaneous cyst aspiration for pain). All other pregnancies carried on to term. Two women required a caesarean section because cysts were obstructing labour. A high proportion of cyst resolution and decrease in size >50% was observed (27/39 simple cysts, 7/9 endometrioma, no dermoids, 8/15 cysts with borderline appearance).

Caspi et al. (2000) followed up 68 pregnancies with ultrasound-diagnosed dermoid cysts smaller than 6 cm, all treated conservatively. Cysts did not increase in size and no cyst accidents were observed. There were no fetal or maternal complications attributable to the presence of dermoid cysts in this group.

Evidence level: 3

Katz et al. (2010) compared outcomes in pregnant women with (n=93) and without ovarian cysts (n=212017). Twenty-two women were diagnosed before or during pregnancy by ultrasound and 71 were diagnosed at CS. Among women with ovarian cysts three cases of ovarian torsion and one of haemorrhage were diagnosed. In the ovarian cyst group 15 women needed hospitalisation due to abdominal pain, but no data is provided for the control group. Fetal outcomes (rate of preterm delivery, low birth weight, APGAR<5 and perinatal mortality) did not differ statistically.

Evidence level: 2-

Majeed et al. (2011) reported 16 women diagnosed with persistent ovarian cysts and followed them up conservatively with ultrasound scan (frequency not stated) until they became symptomatic. Four women had emergency laparotomy due to ovarian cyst torsion and rupture, two women had elective laparotomy in second trimester due to large cyst size (>20 cm) and ten women had a laparotomy in the post- partum period. Fetal outcomes included one miscarriage and all remaining women had a term delivery.

Evidence level: 3

Should the laparoscopic approach to ovarian cysts be preferred over the open approach in pregnancy?

**When surgery is indicated, laparoscopic cystectomy is associated with better maternal outcomes than laparotomy in the second trimester, with similarly good fetal outcomes.**

B

**Good maternal and fetal outcomes have also been reported for laparoscopic surgery for the first and third trimester, however the number of reported cases is small. More research is needed to prove the superiority of one approach over the other outside the second trimester. In the interim, choice of approach should be decided based on local circumstances and expertise.**

✓

A Cochrane database review from 2013 did not identify any RCTs (Bunyavejchevin et al., 2013). A newer systematic review (Liu et al., 2017) of one RCT (Chen et al., 2014), and three non-randomised comparative studies investigated laparoscopic and open surgery for suspected adnexal masses in the second trimester in 240 women. Laparoscopic surgery was associated with a reduced risk of post-operative complications (RR=0.20, CI=0.06–0.72); there was no difference in the risk of post-operative miscarriage (p=0.26). Laparoscopy was associated with lower estimated intra-operative blood loss, lower post-operative pain scores, and a shorter hospitalisation, readmission and immobilisation. Laparoscopy was associated with a longer operation duration compared with laparotomy (mean difference 13.7 min, CI=12.58–14.82, p<0.001). In one of the reviewed studies significantly fewer adhesions at the time of caesarean section were observed in women who had undergone laparoscopy.

Evidence level: 2++

In addition, three further retrospective studies (Carter et al., 2004, Koo et al., 2012 and Martynov et al. 2014) comparing laparoscopic with open approach for persistent or torted ovarian cysts were identified (Koo et al., 2012). These studies confirmed that maternal outcomes were better and fetal outcomes were similar in the laparoscopic compared to the laparotomy group. However, the case number of first and third trimester pregnancies and large cysts (>6 cm) were small. The fact that most reports were retrospective and from specialist centres could lead to the risk of reporting bias, with better outcomes for the reported techniques than could be expected in non-specialist settings.

Evidence level: 2-

**Cyst aspiration with or without concurrent cystectomy may be a safe alternative.**

D

Chung et al. (2001) reported a technique of extra-corporeal cystectomy or oophorectomy after aspiration of the cyst, in four obese second trimester women. Dohi et al. (2012) reports two cases of ultrasound-guided culdotomy for cysts incarcerated in the pouch of Douglas after needle aspiration via scan probe. There were no operative complications and both women had vaginal deliveries. Duic et al. (2002) describes a technique of trans-vaginal ultrasound guided cyst aspiration in 1st and of percutaneous ultrasound guided cyst aspiration in second trimester with good outcomes. Hutt et al. (2000) also reports aspiration of two cases of large ovarian cysts, one twice after re-accumulation with normal pregnancy outcome.

Kitade et al. (2008) reports a 2 puncture extra-corporeal method of cyst aspiration including a specialised retractor and balloon to bring the cyst to a 3 cm suprapubic incision in a case series of n=18.

## 10. Safety of imaging in pregnancy

Guidance on ionising radiation in pregnancy can be found in Health Protections Agency & Royal College of Radiologists & College of Radiographers’ guidance (RCR, 2009). Recommendations on contrast media in pregnancy can be found in the European Society of Urogenital Radiology guidelines (Webb et al., 2005). For the conditions included in this guideline Magnet Resonance Imaging (MRI) with gadolinium is usually not required, since there are alternative modalities, such as MRI without gadolinium and ultrasound. Indications for MRI with gadolinium include the characterization of liver and brain lesions when suspecting a new malignancy, if treatment would make a difference.

The smallest possible dose of one of the most stable gadolinium contrast agents may be given to the pregnant mother if there is a very strong indication for enhanced MRI. No mutagenic and teratogenic effects have been described after maternal administration of gadolinium based on the limited data available (Webb et al., 2005). Webb et al. (2005) states that no additional neonatal tests are required.

## 11. Conclusions

There is mounting evidence that appendicitis, gallbladder disease and symptomatic adnexal cysts can be safely managed laparoscocpically in pregnancy. Most evidence is from specialist centers. Only adequately trained laparoscopists should carry out these procedures and adequate peri- operative preparations are paramount.

Especially outside the second trimester most included papers are from small case series and more high-grade evidence is needed. Because it is difficult to perform RCTs for these conditions, creation and analysis of national large databases appears to be a way forward.

## Appendix 1

Clinical guidelines are systematically developed statements which assist clinicians and women in making decisions about appropriate treatment for specific conditions’. These recommendations are not intended to dictate an exclusive course of management or treatment. They must be evaluated with reference to individual woman’s needs, resources and limitations unique to the institution and variations in local populations. It is hoped that this process of local ownership will help to incorporate these guidelines into routine practice. Attention is drawn to areas of clinical uncertainty where further research may be indicated.

The evidence used in this guideline was graded using the scheme below and the recommendations formulated in a similar fashion with a standardised grading scheme.

### Classification of evidence levels

**1++** High-quality meta-analyses, systematic reviews of randomised controlled trials or randomised controlled trials with a very low risk of bias **1+** Well-conducted meta-analyses, systematic reviews of randomised controlled trials or randomised controlled trials with a low risk of bias **1-** Meta-analyses, systematic reviews of randomised controlled trials or randomised controlled trials with a high risk of bias **2++** High-quality systematic reviews of case– control or cohort studies or high-quality case–control or cohort studies with a very low risk of confounding, bias or chance and a high probability that the relationship is causal **2+** Well-conducted case–control or cohort studies with a low risk of confounding, bias or chance and a moderate probability that the relationship is causal **2-** Case–control or cohort studies with a high risk of confounding, bias or chance and a significant risk that the relationship is not causal **3** Non-analytical studies, e.g. case reports, case series **4** Expert opinion

### Grades of recommendations

**A** At least one meta-analysis, systematic review or randomised controlled trial rated as 1++ and directly applicable to the target population; or A systematic review of randomised controlled trials or a body of evidence consisting principally of studies rated as 1+ directly applicable to the target population and demonstrating overall consistency of results **B** A body of evidence including studies rated as 2++ directly applicable to the target population, and demonstrating overall consistency of results; or Extrapolated evidence from studies rated as 1++ or 1+ **C** A body of evidence including studies rated as 2+ directly applicable to the target population and demonstrating overall consistency of results; or Extrapolated evidence from studies rated as 2++ **D** Evidence level 3 or 4; or Extrapolated evidence from studies rated as 2+

### Good practice point

**✓** Recommended best practice based on the clinical experience of the guideline development group

## References

[1] Abbasi N, Patenaude V, Abenhaim HA (2014). Management and outcomes of acute appendicitis in pregnancy-population-based study of over 7000 cases.. BJOG.

[2] (2017). Nonobstetric surgery during pregnancy. Committee Opinion No. 696. Obstet Gynecol.

[3] Aylin P, Bennett P, Bottle A (2016). Estimating the risk of adverse birth outcomes in pregnant women undergoing non-obstetric surgery using routinely collected NHS data: an observational study..

[4] Balinskaite V, Bottle A, Sodhi V (2017). The Risk of Adverse Pregnancy Outcomes Following Nonobstetric Surgery During Pregnancy: Estimates From a Retrospective Cohort Study of 6.5 Million Pregnancies. Ann Surg.

[5] Balthazar U, Steiner AZ, Boggess JF (2011). Management of a persistent adnexal mass in pregnancy: what is the ideal surgical approach?. J Minim Invasive Gynecol.

[6] Bani Hani MN (2007). Laparoscopic surgery for symptomatic cholelithiasis during pregnancy.. Surg Laparosc Endosc Percutan Tech.

[7] Bhavani-Shankar K, Steinbrook RA, Brooks DC (2000). Arterial to end-tidal carbon dioxide pressure difference during laparoscopic surgery in pregnancy.. Anesthesiology.

[8] Borgfeldt C, Andolf E (1999). Transvaginal sonographic ovarian findings in a random sample of women 25-40 years old.. Ultrasound Obstet Gynecol.

[9] Bunyavejchevin S, Phupong V (2013). Laparoscopic surgery for presumed benign ovarian tumor during pregnancy.. The Cochrane database of systematic reviews.

[10] Candiani M, Maddalena S, Barbieri M (2012). Adnexal masses in pregnancy: fetomaternal blood flow indices during laparoscopic surgery.. J Minimally Invasive Gynecol.

[11] Carter JF, Soper DE (2004). Operative laparoscopy in pregnancy.. JSLS.

[12] Caspi B, Levi R, Appelman Z (2000). Conservative management of ovarian cystic teratoma during pregnancy and labor.. Am J Obstet Gynecol.

[13] Chen L, Ding J, Hua K (2014). Comparative analysis of laparoscopy versus laparotomy in the management of ovarian cyst during pregnancy.. J Obstet Gynaecol Res.

[14] Cheng HT, Wang YC, Lo HC (2015). Laparoscopic appendectomy versus open appendectomy in pregnancy: a population-based analysis of maternal outcome.. Surg Endosc.

[15] Chestnut DH WC, Tsen LC, Ngan Kee WD (2009). Obstetric anesthesia: principles and practice.

[16] Chung JC, Cho GS, Shin EJ (2013). Clinical outcomes compared between laparoscopic and open appendectomy in pregnant women.. Can J Surg.

[17] Chung MK, Chung RP (2001). Laparoscopic extracorporeal oophorectomy and ovarian cystectomy in second trimester pregnant obese patients.. JSLS.

[18] Cluver C, Novikova N, Hofmeyr GJ (2013). Maternal position during caesarean section for preventing maternal and neonatal complications.. Cochrane Database Syst Rev.

[19] Condous G, Okaro E, Bourne T (2003 Oct). The Conservative Management of Early Pregnancy Complications: a Review of the Literature.. Ultrasound Obstet Gynecol.

[20] Cox TC, Huntington CR, Blair LJ (2016). Laparoscopic appendectomy and cholecystectomy versus open: a study in 1999 pregnant patients.. Surg Endosc.

[21] Date RS, Kaushal M, Ramesh A (2008). A review of the management of gallstone disease and its complications in pregnancy.. Am J Surg.

[22] Dohi S, Yamazaki R, Sagawa T (2012). New ultrasound-guided culdotomy of vaginal ovarian cystectomy for pregnant women incarcerated ovarian dermoid cysts in recto-uterine pouch: case reports.. Endoscopy.

[23] Duic Z, Kukura V, Ciglar S (2002). Adnexal masses in pregnancy: a review of eight cases undergoing surgical management.. European journal of gynaecological oncology.

[24] Erekson EA, Brousseau EC, Dick-Biascoechea MA (2012). Maternal postoperative complications after nonobstetric antenatal surgery.. J Matern Fetal Neonatal Med.

[25] Friedman JD, Ramsey PS, Ramin KD (2002). Pneumoamnion and pregnancy loss after second-trimester laparoscopic surgery.. Obstet Gynecol.

[26] Hasson J, Tsafrir Z, Azem F (2010). Comparison of adnexal torsion between pregnant and nonpregnant women.. Am J Obstet Gynecol.

[27] Holzer T, Pellegrinelli G, Morel P (2011). Appendectomy during the third trimester of pregnancy in a 27-year old patient: case report of a “near miss” complication.. Patient Saf Surg.

[28] Hutt R, Long M, Sturdy J (2000). Conservative management of ovarian cysts in pregnancy during third trimester and intrapartum. J Obstet Gynaecol.

[29] Jeong JS, Ryu DH, Yun HY (2011). Laparoscopic appendectomy is a safe and beneficial procedure in pregnant women.. Surg Laparosc Endosc Percutan Tech.

[30] Joumblat N, Grubbs B, Chmait RH (2012). Incidental fetoscopy during laparoscopy in pregnancy: management of perforation of the gravid uterus.. Surg Laparosc Endosc Percutan Tech.

[31] Katz L, Levy A, Wiznitzer A (2010). Pregnancy outcome of patients with dermoid and other benign ovarian cysts.. Arch Gynecol Obstet.

[32] Kirshtein B, Perry ZH, Avinoach E (2009). Safety of laparoscopic appendectomy during pregnancy.. World J Surg.

[33] Kitade M, Takeuchi H, Kumakiri J (2008). Instruments and Techniques: Two-Puncture Extracorporeal Method—a New Technique for Laparoscopic Management of Ovarian Tumors Detected During Pregnancy.. J Gynecol Surg.

[34] Koo YJ, Lee JE, Lim KT (2011a). A 10-year experience of laparoscopic surgery for adnexal masses during pregnancy.. Int J Gynaecol Obstet.

[35] Koo YJ, Kim TJ, Lee JE (2011b). Risk of torsion and malignancy by adnexal mass size in pregnant women.. Acta Obstet Gynecol Scand.

[36] Koo YJ, Kim HJ, Lim KT (2012). Laparotomy versus laparoscopy for the treatment of adnexal masses during pregnancy.. Aust N Z J Obstet Gynaecol.

[37] Kuczkowski KM (2006). The safety of anaesthetics in pregnant women.. Expert Opin Drug Saf.

[38] Kuy S, Roman SA, Desai R (2009). Outcomes following cholecystectomy in pregnant and nonpregnant women.. Surgery.

[39] Laustsen JF, Bjerring OS, Johannessen O (2016). Laparoscopic appendectomy during pregnancy is safe for both the mother and the fetus.. Dan Med J.

[40] Lee GS, Hur SY, Shin JC (2004). Elective vs. conservative management of ovarian tumors in pregnancy. Int J Gynaecol Obstet.

[41] Lee YY, Kim TJ, Choi CH (2010). Factors influencing the choice of laparoscopy or laparotomy in pregnant women with presumptive benign ovarian tumors.. Int J Gynaecol Obstet.

[42] Lenglet Y, Roman H, Rabishong B (2006). Laparoscopic management of ovarian cysts during pregnancy.. Gynecol Obstet Fertil.

[43] Liu YX, Zhang Y, Huang JF (2017). Meta-analysis comparing the safety of laparoscopic and open surgical approaches for suspected adnexal mass during the second trimester.. Int J Gynaecol Obstet.

[44] Lyass S, Pikarsky A, Eisenberg VH (2001). Surg Endosc. Is laparoscopic appendectomy safe in pregnant women.

[45] Machado NO, Machado LS (2009). Laparoscopic cholecystectomy in the third trimester of pregnancy: report of 3 cases.. Surg Laparosc Endosc Percutan Tech.

[46] Majeed T, Sattar Y, Waheed F (2011). Management of Ovarian Cyst in Pregnancy and its Effect on Pregnancy Outcome.. PJMHS.

[47] Martynov SA, Adamyan LV, Danilov AU (2014). Comparison of Laparoscopy and Laparotomy in Treatment of Adnexal Masses during Pregnancy. JMIG.

[48] Mathevet P, Nessah K, Dargent D (2003). Laparoscopic management of adnexal masses in pregnancy: a case series.. Eur J Obstet Gynecol Reprod Biol.

[49] Mazze RI, Kallen B (1989). Reproductive outcome after anesthesia and operation during pregnancy: a registry study of 5405 cases.. Am J Obstet Gynecol.

[50] McGory ML, Zingmond DS, Tillou A (2007). Negative appendectomy in pregnant women is associated with a substantial risk of fetal loss.. J Am Coll Surg.

[51] Miloudi N, Brahem M, Ben Abid S (2012). Acute appendicitis in pregnancy: specific features of diagnosis and treatment.. Journal of visceral surgery.

[52] Minig L, Otano L, Cruz P (2016). Laparoscopic surgery for treating adnexal masses during the first trimester of pregnancy.. J Minim Access Surg.

[53] Mourad J, Elliott JP, Erickson L (2000). Appendicitis in pregnancy: new information that contradicts long-held clinical beliefs.. Am J Obstet Gynecol.

[54] Nasioudis D, Tsilimigras D, Economopoulos KP (2016). Laparoscopic cholecystectomy during pregnancy: A systematic review of 590 patients.. Int J Surg.

[55] (2015). Ectopic pregnancy and miscarriage: diagnosis and initial management. NICE clinical guideline [CG154].

[56] (2011). Ovarian Cancer: The Recognition and Initial Management of Ovarian Cancer. NICE clinical guideline [CG122].

[57] (2015). Preterm labour and birth. NICE guideline [NG25].

[58] Othman MO, Stone E, Hashimi M (2012). Conservative management of cholelithiasis and its complications in pregnancy is associated with recurrent symptoms and more emergency department visits.. Gastrointest Endosc.

[59] Park SH, Park MI, Choi JS (2010). Laparoscopic appendectomy performed during pregnancy by gynecological laparoscopists.. Eur J Obstet Gynecol Reprod Biol.

[60] Peng P, Zhu L, Lang JH (2013). Clinical analysis of laparoscopic surgery for ovarian masses under different conditions during the second trimester.. Chin Med J.

[61] Qureshi H, Massey E, Kirwan D (2014). BCSH guideline for the use of anti-D immunoglobulin for the prevention of haemolytic disease of the fetus and newborn.. Transfusion Medicine.

[62] Rajmohan N, Prakasam H, Simy J (2013). Laparoscopic surgeries during second and third trimesters of pregnancy in a tertiary care centre in South India: Anaesthetic implications and long- term effects on children.. Indian J Anaesth.

[63] (2009). Protection of Pregnant Patients during Diagnostic Medical Exposures to Ionising Radiation. Advice from the Health Protection Agency.

[64] Reedy MB, Kallen B, Kuehl TJ (1997). Laparoscopy during pregnancy: a study of five fetal outcome parameters with use of the Swedish Health Registry.. Am J Obstet Gynecol.

[65] Rizzo AG (2003). Laparoscopic surgery in pregnancy: long-term follow-up.. J Laparoendosc Adv Surg Tech A.

[66] (2008). Preventing Entry-related Gynaecological Laparoscopic Injuries. Green-top Guideline No 49.

[67] (2011). Management of Suspected Ovarian Masses in Premenopausal Women. Green-top Guideline No 62.

[68] (2015). Thrombosis and Embolism during Pregnancy and the Puerperium, Reducing the Risk. Green-top Guideline No 37a.

[69] (2015). Thrombosis and Embolism during Pregnancy and the Puerperium, the Acute Management of. Green-top Guideline No 37b.

[70] Scheib SA, Jones HH, Boruta DM (2013). Laparoendoscopic single-site surgery for management of adnexal masses in pregnancy: case series.. J Minim Invasive Gynecol.

[71] Schirmer BD, Winters KL, Edlich RF (2005). Cholelithiasis and cholecystitis.. J Long Term Eff Med Implants.

[72] Sedaghat N, Cao AM, Eslick GD (2017). Laparoscopic versus open cholecystectomy in pregnancy: a systematic review and meta-analysis.. Surg Endosc.

[73] Segev L, Segev Y, Rayman S (2016). Appendectomy in Pregnancy: Appraisal of the Minimally Invasive Approach.. J Laparoendosc Adv Surg Tech A.

[74] Silvestri MT, Pettker CM, Brousseau EC (2011). Morbidity of appendectomy and cholecystectomy in pregnant and nonpregnant women.. Obstet Gynecol.

[75] Steinbrook RA, Brooks DC, Datta S (1996). Laparoscopic cholecystectomy during pregnancy. Review of anesthetic management, surgical considerations. Surg Endosc.

[76] Struthers AD, Cuschieri A (1998). Cardiovascular consequences of laparoscopic surgery.. Lancet.

[77] Sungler P, Heinerman PM, Steiner H (2014). Laparoscopic cholecystectomy and interventional endoscopy for gallstone complications during pregnancy.. Surg Endosc.

[78] Swisher SG, Schmit PJ, Hunt KK (1994). Biliary disease during pregnancy.. Am J Surg.

[79] Takeda A, Hayashi S, Imoto S (2014). Pregnancy outcomes after emergent laparoscopic surgery for acute adnexal disorders at less than 10 weeks of gestation.. J Obstet Gynaecol Res.

[80] Tamir IL, Bongard FS, Klein SR (1990). Acute appendicitis in the pregnant patient.. Am J Surg.

[81] Tsafrir Z, Hasson J, Levin I (2012). Adnexal torsion: cystectomy and ovarian fixation are equally important in preventing recurrence.. Eur J Obstet Gynecol Reprod Biol.

[82] Upadhyay A, Stanten S, Kazantsev G (2007). Laparoscopic management of a nonobstetric emergency in the third trimester of pregnancy.. Surg Endosc.

[83] Walsh CA, Tang T, Walsh SR (2008). Laparoscopic versus open appendicectomy in pregnancy: a systematic review.. Int J Surg.

[84] Webb JA, Thomsen HS, Morcos SK (2005). The use of iodinated and gadolinium contrast media during pregnancy and lactation.. Eur Radiol.

[85] Weiner E, Mizrachi Y, Keidar R (2015). Laparoscopic surgery performed in advanced pregnancy compared to early pregnancy.. Arch Gynecol Obstet.

[86] Wilasrusmee C, Sukrat B, McEvoy M (2012). Systematic review and meta-analysis of safety of laparoscopic versus open appendicectomy for suspected appendicitis in pregnancy.. Br J Surg.

[87] Yuen PM, Ng PS, Leung PL (2004). Outcome in laparoscopic management of persistent adnexal mass during the second trimester of pregnancy.. Surg Endosc.

[88] Yuk JS, Kim YJ, Hur JY (2013). Association between pregnancy and acute appendicitis in South Korea: a population-based, cross-sectional study.. J Korean Surg Soc.

[89] Zanetta G, Mariani E, Lissoni A (2003). A prospective study of the role of ultrasound in the management of adnexal masses in pregnancy.. BJOG.

