# Correction: Evidence-Based Guideline on Laparoscopy in Pregnancy: Commissioned by the British Society for Gynaecological Endoscopy (BSGE) Endorsed by the Royal College of Obstetricians & Gynaecologists (RCOG)

**Published:** 2020-01-24

**Authors:** E Ball, N Waters, N Cooper, C Talati, R Mallick, S Rabas, A Mukherjee, Y Sri Ranjan, M Thaha, R Dodia, R Keedwell, M Madhra, N Kuruba, R Malhas, E Gaughan, K Tompsett, H Gibson, H Wright, C Gnanachandran, L Gokhale, T Hookaway, C Baker, K Murali, D Jurkovic, N Amso, J Clark, S Thangaratinam, T Chalhoub, P Kaloo, E Saridogan

**Affiliations:** Royal London Hospital;; Royal Surrey County Hospital NHS Trust;; Homerton Hospital;; Brighton and Sussex University Hospitals NHS Trust;; Queen’s Hospital London and King George Hospital;; Northwick Park Hospital;; Chelsea and Westminster Hospital;; John Radcliffe Hospital;; Royal Cornwall Hospitals NHS Trust;; Royal Infirmary of Edinburgh;; Norfolk and Norwich University Hospital;; Walsall Manor Hospital;; Rotunda Hospital, Dublin;; Barking, Havering and Redbridge University Hospitals NHS Trust;; North Manchester General Hospital;; Northampton General Hospital;; Royal Gwent Hospital Aneurin Bevan Health Board;; Plymouth Hospitals NHS Trust;; Poole Hospital;; Salisbury District and General Hospital;; University College Hospital;; Cardiff University School of Medicine;; Birmingham Women’s Hospital;; Royal Victoria Infirmary;; Gloucestershire Hospitals NHS Foundation Trust.

The name of the tenth author was spelled incorrectly. The correct name is R Dodia.

One author was missing and has been added: L Gokhale.

The full corrected article can be downloaded here: http://fvvo.be/assets/802/FVVinObGyn-11-5-r1.pdf

